# Mapping Cancer in Africa: A Comprehensive and Comparable Characterization of 34 Cancer Types Using Estimates From GLOBOCAN 2020

**DOI:** 10.3389/fpubh.2022.839835

**Published:** 2022-04-25

**Authors:** Rajesh Sharma, Aashima, Mehak Nanda, Claudio Fronterre, Paul Sewagudde, Anna E. Ssentongo, Kelsey Yenney, Nina D. Arhin, John Oh, Forster Amponsah-Manu, Paddy Ssentongo

**Affiliations:** ^1^University School of Management and Entrepreneurship, Delhi Technological University, New Delhi, India; ^2^Centre for Health Informatics, Computing, and Statistics, Lancaster University, Lancaster, United Kingdom; ^3^Relife Family Medical Center, Kampala, Uganda; ^4^Department of Public Health Sciences, Penn State College of Medicine, Hershey, PA, United States; ^5^Division of Trauma Surgery, Department of Surgery, Penn State College of Medicine and Milton S. Hershey Medical Center, Hershey, PA, United States; ^6^Washington State University Elson S. Floyd College of Medicine, Seattle, WA, United States; ^7^Division of Hematology/Oncology, Department of Medicine, Vanderbilt University Medical Center, Nashville, TN, United States; ^8^Department of Surgery, Eastern Regional Hospital, Koforidua, Ghana; ^9^Center for Neural Engineering, The Pennsylvania State University, State College, PA, United States

**Keywords:** cancer burden, mapping, spatial epidemiology, incidence, mortality, Africa, GLOBOCAN

## Abstract

**Objective:**

Cancer incidence and mortality rates in Africa are increasing, yet their geographic distribution and determinants are incompletely characterized. The present study aims to establish the spatial epidemiology of cancer burden in Africa and delineate the association between cancer burden and the country-level socioeconomic status. The study also examines the forecasts of the cancer burden for 2040 and evaluates infrastructure availability across all African countries.

**Methods:**

The estimates of age, sex, and country-specific incidence and mortality of 34 neoplasms in 54 African countries, were procured from GLOBOCAN 2020. Mortality-to-incidence ratio (MIR) was employed as a proxy indicator of 5-year survival rates, and the socioeconomic development of each country was measured using its human development index (HDI). We regressed age-standardized incidence rate (ASIR), age-standardized mortality rate (ASMR), and MIR on HDI using linear regression model to determine the relationship between cancer burden and HDI. Maps were generated for each cancer group for each country in Africa. The data about the cancer infrastructure of African countries were extracted from the WHO Cancer Country Profiles.

**Results:**

In Africa, an estimated 1.1 million new cases [95% uncertainty intervals (UIs) 1.0 – 1.3 million] and 711,429 [611,604 – 827,547] deaths occurred due to neoplasms in 2020. The ASIR was estimated to be 132.1/100,000, varying from 78.4/100,000 (Niger) to 212.5/100,000 (La Réunion) in 2020. The ASMR was 88.8/100,000 in Africa, ranging from 56.6/100,000 in the Republic of the Congo to 139.4/100,000 in Zimbabwe. The MIR of all cancer combined was 0.64 in Africa, varying from 0.49 in Mauritius to 0.78 in The Gambia. HDI had a significant negative correlation with MIR of all cancer groups combined and main cancer groups (prostate, breast, cervical and colorectal). HDI explained 75% of the variation in overall 5-year cancer survival (MIR). By 2040, the burden of all neoplasms combined is forecasted to increase to 2.1 million new cases and 1.4 million deaths in Africa.

**Conclusion:**

High cancer mortality rates in Africa demand a holistic approach toward cancer control and management, including, but not limited to, boosting cancer awareness, adopting primary and secondary prevention, mitigating risk factors, improving cancer infrastructure and timely treatment.

## Introduction

Africa is undergoing significant improvements in population health, which is evident from declining infant mortality rates, plummeting HIV/AIDS fatality rates, and rising life spans (1, 2). These gains are attributable to improved vaccination coverage (3), malaria interventions such as bed nets (4), antiretroviral therapy for HIV, and prevention of mother-to-child transmission of HIV (5).

Amidst these gains, the disease landscape in Africa is also undergoing significant changes, with rising morbidity and mortality due to non-infectious diseases (for example, cancer, cardiovascular disease, type 2 diabetes) and falling burden of communicable diseases (1). Cancer is now the fifth leading cause of death in Africa (1), which warrants further investigation about cancer burden in African region. Previous studies on cancer epidemiology in Africa focussed on sub-Saharan Africa (6), or individual cancer group such as childhood cancer (7), cervical cancer (8), breast cancer (9, 10), prostate cancer (11), colorectal cancer (12, 13), and the role of infections in cancer pathogenesis (14). A previous study examined the burden of cancer in Africa but was based upon GLOBOCAN 2012 (15) database. However, no previous study provides the comprehensive epidemiology of different cancer groups in all countries of Africa and estimates the relationship between human development index (HDI) and cancer burden in Africa.

In the present study, we aimed to examine cancer burden in Africa using the recently released GLOBOCAN 2020 estimates (16, 17). First, we examined the estimates of age, sex and country-specific incidence and mortality of 34 cancer groups in 54 African countries. Second, we investigated the association between leading cancer groups and country-level socioeconomic status measured by HDI (18). Third, to provide a comparative perspective, we compared the incidence, death counts, and age-standardized rates of leading cancer groups in Africa with other continents. Fourth, we examined the forecasted estimates of the cancer burden in Africa for all neoplasms by 2040. Lastly, we examined the availability of cancer infrastructure in African countries. This comprehensive examination of recent cancer burden and their age-standardized rates along with their association with country-level socioeconomic status, can guide the formulation and implementation of public health policies to prevent, screen, and treat cancer in Africa.

## Methods

### Overview of GLOBOCAN 2020 Estimates

The estimates of incidence and mortality for 34 cancer groups in 54 African countries were retrieved from GLOBOCAN 2020 (16, 17, 19). All 34 cancer groups, along with International Classification of Diseases codes, are presented in Supplementary Table 1. In 2020, the International Agency for Research on Cancer (IARC) in its recent GLOBOCAN iteration produced age, sex and country-specific estimates for 2020 in 18 non-overlapping age-groups: under-5, 5–9, 10–14, 15–19, 20–24, 25–29, 30–34, 35–39, 40–44, 45–49, 50–54, 55–59, 60–64, 65–69, 70–74, 75–79, 80–84, and 85 plus. Below we describe GLOBOCAN 2020 methodology to arrive at country-level estimates.

### GLOBOCAN Methodology

In GLOBOCAN 2020, for estimation of incidence and mortality, different methods for different set of countries were employed (16, 17, 20) as per the availability of the historical national data, and national or regional registries. In method 1, incidence rates of 2020 were predicted using short term prediction model by Dyba and Hakulinen (21) for 45 countries for which at least 6–10 years of national cancer incidence data were available. Method 2 was used for countries in which no historical national cancer incidence and mortality data were available. For 54 countries, most recent incidence rates (national (2a) or regional (2b) were used as a proxy for 2020. In case national mortality data were available and registries were subnational, method 3a and 3b were applied. In method 3a, incidence rates were estimated using mortality-to-incidence ratios (MIRs) for 14 countries, which were derived from cancer registries of those countries. In method 3b, for 37 countries, incidence rates were estimated using MIR from neighboring countries and the MIR was scaled as per HDI status of a country. According to method 4, when national or subnational registries were not available and mortality data were also not available and within country source information wasn't accurate, age and sex-wise national incidence rates for all cancers combined were obtained by averaging overall rates from neighboring countries. The site-specific cancer rates for all cancers combined were obtained from overall rates using cancer-specific relative frequency data. For five countries, method 4 was applied. Method 9 was applied for 30 countries for which neither national/regional registries, national mortality data nor within country source information were available. Under this method, average incidence rates from selected neighboring countries were used as incidence rates.

For estimation of mortality, under method 1 (when historical national mortality data were available), short term prediction models were applied to obtain estimates for the year 2020 for 80 countries. For 21 countries, where national mortality data were available from national or subnational sources, the most recently observed mortality rates (national (2a) or regional (2b) were applied to the 2020 population. For 81 countries, recent mortality data were not available; for these countries, method 3 was applied. Accordingly, national mortality rates were estimated from national incidence and fitted incidence-to-mortality (IM) ratios derived from cancer registries in neighboring countries, and IM ratios were scaled as per country-level HDI. In countries where recent mortality data were unavailable and IM ratios couldn't be calculated as in method 3, for such three countries, method 9 was used in which rates were estimated as an average of those from selected neighboring countries (22).

We used the MIR as a proxy of 5-year survival rates, which has also been considered a proxy indicator of the 5-year survival rate of various neoplasms (23–25). MIR was calculated by dividing all-age death counts by all-age new cases. In the present study, all-age, age-specific, and sex-specific incidence and death rates are reported along with 95% uncertainty intervals (UIs); the UIs reflect uncertainties associated with cancer registry coverage and death registration, data quality, and timeliness of data reporting (16, 17). GLOBOCAN provided the projected number of cases and deaths for the year 2040 (16, 26). The forecasted incidence and mortality rates for cancer in Africa in 2040 are procured from Global Cancer Observatory (26) maintained by the IARC. The estimates of 2040 were produced by Sung et al. (16) by applying the incidence and death rates of 2020 to the United Nations population estimates for 2040. These 2040 estimates of cancer-specific incidence and death counts are based on the assumption that risk factors and rates of cancer incidence and death remain unchanged between 2020 and 2040.

The progress of a country against cancer and its inter-linkage with country-level development was gauged by socioeconomic status measured by its HDI (18, 27). HDI is a composite indicator of development, comprising gross national income per capita, education (mean years of schooling and expected years of schooling), and life expectancy at birth, and is calculated as the geometric mean of these three indicators. To examine the relationship between cancer burden and HDI, we have used Pearson's correlation coefficient and regressed age-standardized incidence rate (ASIR), age-standardized mortality rate (ASMR) and MIR on HDI in the linear form.

The data about the cancer infrastructure in African countries were extracted from the WHO Cancer Country Profiles (28). All the maps were produced with the R software for statistical computing version 3.6.3. The African shapefile was retrieved from the spData package (version 0.3.0, https://CRAN.R-project.org/package=spData) and the maps were created with the tmap package (version 3.2.2, https://CRAN.R-project.org/package=tmap).

## Results

### Aggregate Cancer Burden in Africa

In Africa, 1.1 million [95% UI, 1.0 – 1.3 million] new cases and 711,429 [611,604 – 827,547] deaths were estimated in 2020 (Table 1). The overall (male + female) ASIR and ASMR was 132.1/100,000 and 88.8/100,000, respectively (Table 1). Females accounted for 633,456 [525,277 – 763,915] cases and 387,546 [316,060 – 475,200] deaths in comparison to males (incidence: 475,753 [386,823 – 585,128]; deaths: 323,883 [258,540 – 405,740]) (Supplementary Table 2). The ASIR in males was estimated to be 126.8/100,000, lower than females (139.5/100,000). In terms of ASMR, there was lesser disparity between males and females (male: 90.4/100,000; female: 89.2/100,000). The overall MIR for Africa was 0.64, with male MIR of 0.68 and female MIR of 0.61 (Table 1, Supplementary Table 2).

**Table 1:** Aggregate cancer burden in 54 African countries in 2020.

**Population**	**Incidence [95% UI]**	**ASIR**	**ASMR**	**Mortality [95% UI]**	**MIR**
Algeria	58,418 [56,916–59,960]	135.3	76.1	32,802 [31,875–33,756]	0.56
Angola	20,327 [18,854–21,915]	130.6	86.5	12,599 [11,627–13,652]	0.62
Benin	6,747 [5,981–7,610]	95.8	69.3	4,662 [4,065–5,347]	0.69
Botswana	2,010 [1,874–2,156]	109.5	63.4	1,112 [1,036–1,193]	0.55
Burkina Faso	12,045 [10,722–13,531]	112	84.4	8,695 [7,649–9,884]	0.72
Burundi	7,929 [7,038–8,932]	135.5	103.8	5,701 [5,060–6,422]	0.72
Cabo Verde	825 [723–941]	179	107.6	488 [428–557]	0.59
Cameroon	20,745 [19,144–22,480]	127.6	86.6	13,199 [12,132–14,359]	0.64
Central African Republic	2,675 [2,270–3,152]	100.4	76.8	1,957 [1,676–2,285]	0.73
Chad	8,575 [7,277–10,104]	102.3	77.3	6,083 [5,210–7,103]	0.71
Comoros	609 [541–686]	111.8	79	411 [365–463]	0.67
Republic of the Congo	2,478 [2,242–2,739]	84.4	56.6	1,595 [1,428–1,781]	0.64
Côte d'Ivoire	17,300 [16,454–18,189]	123.3	88	11,760 [11,128–12,428]	0.68
Democratic Republic of Congo	48,839 [41,448–57,548]	102.9	74.9	34,412 [29,472–40,180]	0.7
Djibouti	765 [679–862]	91.0	65.3	527 [468–594]	0.69
Egypt	134,632 [131,480–137,860]	159.4	108.6	89,042 [86,773–91,371]	0.66
Equatorial Guinea	927 [787–1,092]	110.1	76.2	592 [507–691]	0.64
Eritrea	2,408 [2,137–2,713]	100.8	72.1	1,670 [1,482–1,881]	0.69
Eswatini	992 [917–1,074]	127.9	82.2	613 [560–671]	0.62
Ethiopia	77,352 [73,647–81,243]	106.7	75.3	51,865 [49,048–54,844]	0.67
France, La Réunion	2,965 [2,815–3,123]	212.5	97.8	1,523 [1,411–1,644]	0.51
Gabon	1,750 [1,516–2,020]	115.8	71.5	1,030 [882–1,203]	0.59
Ghana	24,009 [21,821–26,416]	115.9	80.6	15,802 [14254–17518]	0.66
Guinea	7,871 [7,297–8,490]	116.6	90.7	5,888 [5,465–6,344]	0.75
Guinea–Bissau	1,127 [902–1,408]	107.2	83.3	836 [679–1,029]	0.74
Kenya	42,116 [40,219–44,103]	149.2	103.2	27,092 [25,708–28,550]	0.64
Lesotho	1,876 [1,711–2,057]	109	74.8	1,256 [1,148–1,374]	0.67
Liberia	3,552 [2,842–4,439]	119.3	90.5	2,603 [2,114–3,205]	0.73
Libya	7,661 [7,063–8,309]	132.2	88.2	4,750 [4,381–5,150]	0.62
Madagascar	20,681 [18,358–23,298]	124.3	87.7	13,837 [12,282–15,588]	0.67
Malawi	17,936 [16,569–19,416]	154.2	113.4	12,454 [11,444–13,553]	0.69
Mali	14,185 [13,301–15,127]	140.3	107.5	10,234 [9,540.9–10,977]	0.72
Mauritania	3,079 [2,464–3,848]	108.6	78.3	2,121 [1,723–2,611]	0.69
Mauritius	3,050 [2,910–3,197]	155	72.4	1,504 [1,400–1,615]	0.49
Morocco	59,370 [57,415–61,392]	148.3	87.9	35,265 [34,015–36,561]	0.59
Mozambique	25,446 [23,874–27,122]	135.3	100.4	18,014 [16,766–19,355]	0.71
Namibia	3,345 [3,199–3,497]	198.3	116.1	1,876 [1,783–1,974]	0.56
Niger	9,787 [8,054–11,893]	78.4	62.2	7,382 [5,995–9,089]	0.75
Nigeria	124,815 [118,101–131,911]	110.4	74.8	78,899 [74,234–83,857]	0.63
Rwanda	8,835 [7,948–9,821]	113.9	81.4	6,044 [5,365–6,809]	0.68
Sao Tome and Principe	151 [69–328]	126.4	84.5	103 (47–224)	0.68
Senegal	11,317 [9,056–14,143]	119.5	87.4	7,893 [6,410–9,718]	0.7
Sierra Leone	4,708 [4,579–4,840]	102.1	76.9	3,389 [3,288–3,493]	0.72
Somalia	10,134 [8,995–11,417]	118.1	90.9	7,439 [6,603–8,380]	0.73
South Africa	108,168 [107,065–109,282]	209.5	111.7	56,802 [56,187–57,423]	0.53
South Sudan	6,312 [5,603–7,111]	94.7	72.2	4,633 [4,112–5,219]	0.73
Sudan	27,382 [26,675–28,108]	95.7	63.2	17,055 [16,600–17,523]	0.62
Tanzania	40,464 [38,682–42,328]	133.7	94.3	26,945 [25,586–28,376]	0.67
The Gambia	1,035 [904–1,184]	79.5	65.7	810 [706–930]	0.78
Togo	5,208 [4,765–5,693]	111.7	79.1	3,468 [3,176–3,787]	0.67
Tunisia	19,446 [18,737–20,182]	133.5	78.7	11,855 [11,404–12,323]	0.61
Uganda	34,008 [32,620–35,455]	153.8	112.4	22,992 [21,938–24,096]	0.68
Zambia	13,831 [13,007–14,708]	153.5	103.3	8,672 [8,086–9,300]	0.63
Zimbabwe	16,083 [15,394–16,802]	200.4	139.4	10,676 [10,163–11,215]	0.66
Africa	1,109,209 [965,409–1,274,430]	132.1	88.8	711,429 [611,604–827,547]	0.64

*Incidence, all-age new cases; Mortality, all-age deaths; ASIR, age-standardized incidence rate (cases per 100,000); ASMR, age-standardized mortality rate (deaths per 100,000); MIR, mortality-to-incidence ratio. Data source: GLOBOCAN 2020 (International Agency for Research on Cancer). The figures inside square brackets depict 95% uncertainty intervals*.

### Country-Specific Cancer Burden

Egypt was the leading country with 134,632 [131,480 – 137,860] new cases and 89,042 [86,773 – 91,371] deaths (Table 1); Nigeria was ranked second, followed by South Africa, with an estimated incidence of 124,815 [118,101 – 131,911] and 108,168 [107,065 – 109,282]), respectively. Egypt 89,042 [86,773-91,371], Nigeria 78,899 [74,234 – 83,857], and South Africa 56,802 [56,187 – 57,423] were the top three ranked countries in terms of cancer deaths. In terms of ASIR, La Réunion (212.5/100,000), South Africa (209.5/100,000), and Zimbabwe (200.4/100,000) were the three leading countries, and the ASIR was the lowest in the Gambia (79.5/100,000) and Niger (78.4/100,000) (Table 1). ASMR was highest in Zimbabwe (139.4/100,000), followed by Namibia (116.1/100,000) and lowest in Republic of the Congo (56.6/100,000) (Table 1). MIR was lowest (highest 5-year survival rate) in Mauritius (0.49), La Réunion (0.51), and South Africa (0.53) and highest (worst 5-year survival rates) in Guinea (0.75), Niger (0.75), and The Gambia (0.78).

### Main Cancers in Africa

**Figure 1:**
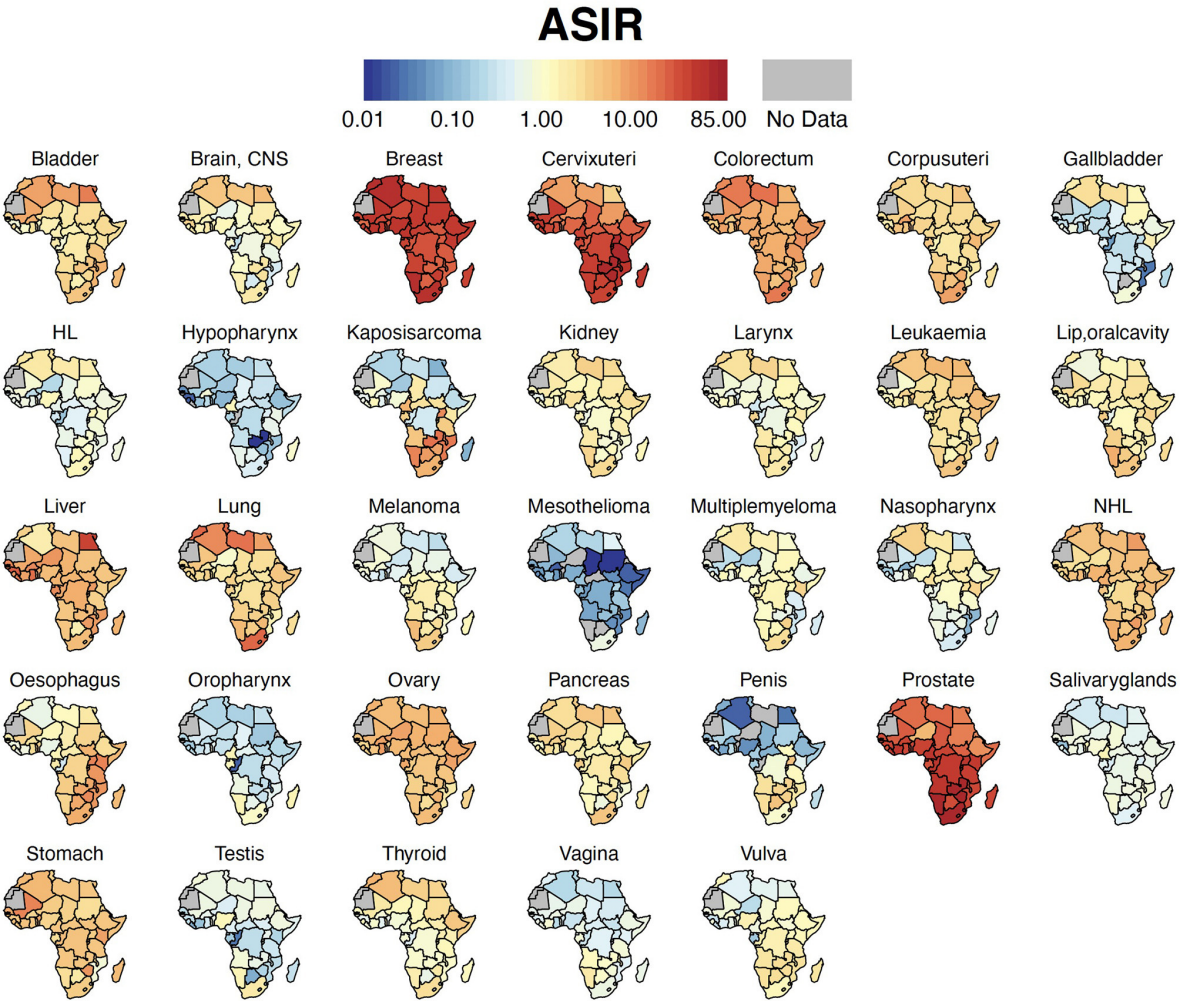
Country-specific ASIR. Cancer ASIR per 100,000 for each country in Africa. ASIR, Age-standardized Incidence Rate; HL, Hodgkin Lymphoma; NHL, Non-Hodgkin Lymphoma; CNS, Central Nervous System.

**Figure 2:**
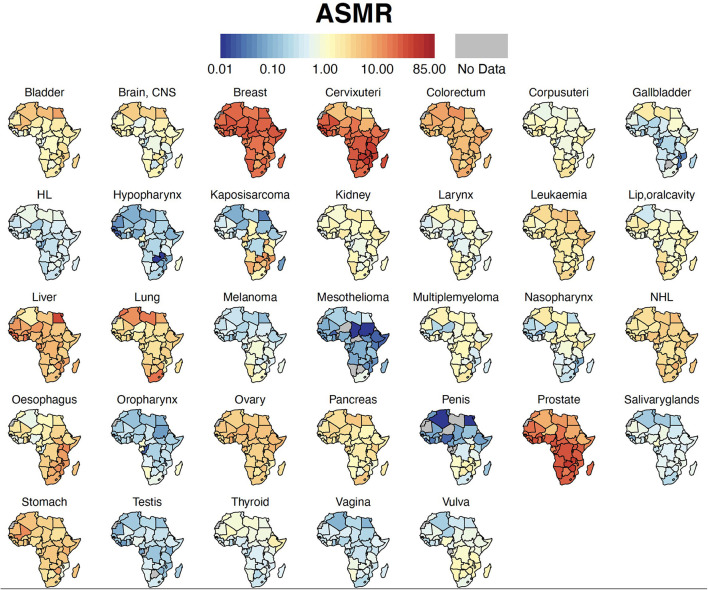
Country-specific ASMR. Cancer ASMR per 100,000 for each country in Africa. ASMR, Age-standardized Mortality Rate; HL, Hodgkin Lymphoma; NHL, Non-Hodgkin Lymphoma, CNS, Central Nervous System.

**Figure 3:**
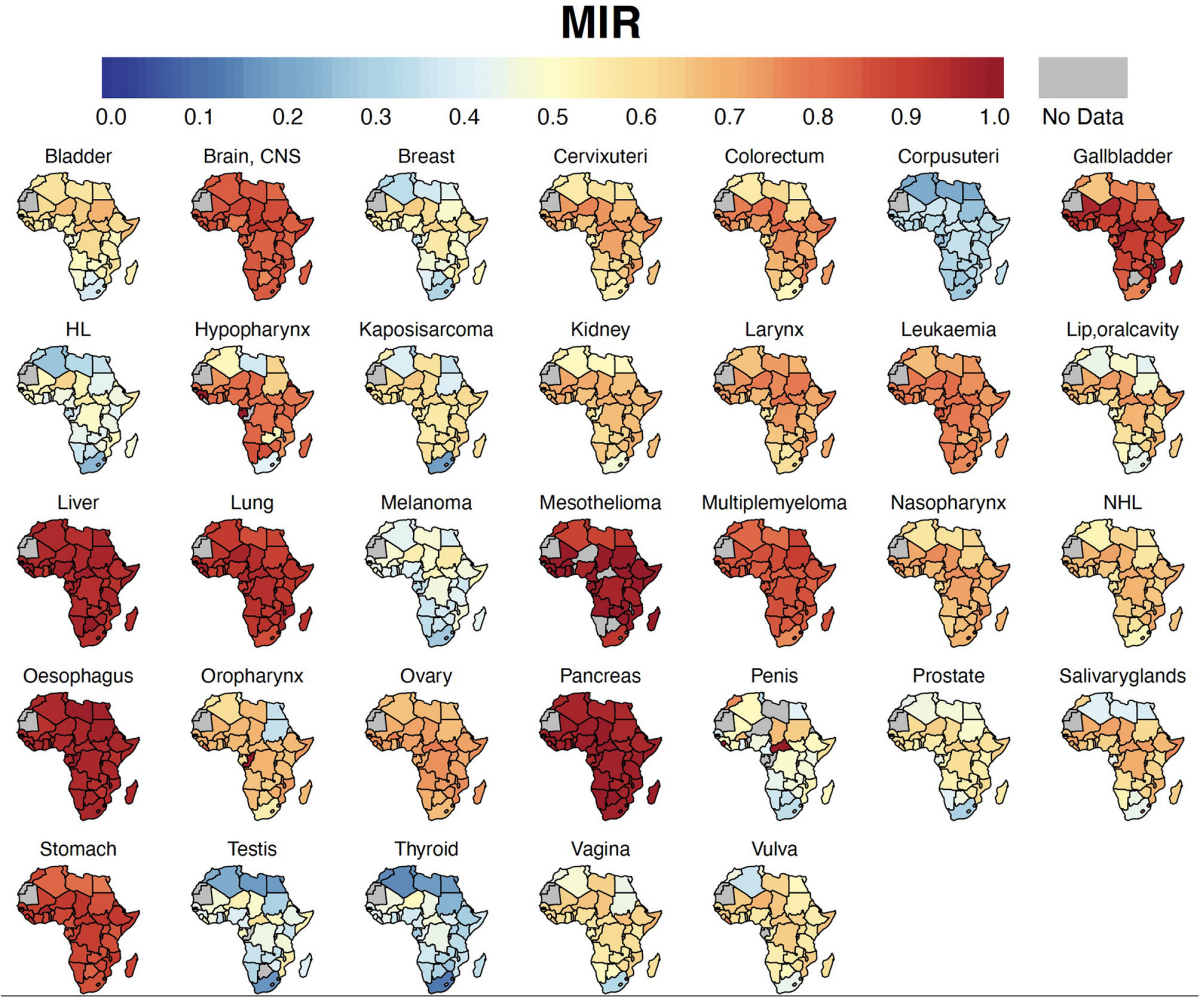
Country-specific MIR. Cancer MIR (proxy for 5-year survival rate) for each country in Africa. MIR, Mortality-to-incidence ratio; HL, Hodgkin Lymphoma; NHL, Non-Hodgkin Lymphoma; CNS, Central Nervous System.

Breast cancer was the leading malignancy in females in Africa, with 186,598 [173,041 – 201,217] new cases and 85,787 [77,648 – 94,779] deaths in 2020 (Table 2). Cervix uteri (cervical cancer, henceforth) was ranked second in Africa with an estimated incidence of 117,316 [105,999 – 129,842] and 76,745 [68,380 – 86,133] deaths. Prostate cancer was the leading cancer group in African males with 93,173 [83,906 – 103,463] cases and 47,249 [41,941 – 53,228] deaths (Table 2). Geographical maps of cancer and country-specific ASIR, ASMR, and MIR are given in Figures 1–3, respectively. All the countries in North Africa (Algeria, Egypt, Libya, Morocco, Sudan, and Tunisia) had greater ASIR and ASMR of breast cancer than cervical cancer (Figures 1, 2); reverse was true in majority sub-Saharan African countries (Supplementary Figures 1, 2). Cervical cancer was not among the top 10 cancer groups in Egypt. Notably, breast cancer, cervical cancer, and prostate cancer were among the top-3 cancer groups in most African countries (Figures 1–3, Supplementary Figures 1, 2).

**Table 2:** Cancer-specific burden of 34 cancer groups in Africa, 2020 (both sexes combined).

**Cancer**	**Incidence [95% UI]**	**Mortality [95% UI]**	**ASIR**	**ASMR**	**MIR**
Bladder	33,196 [27,881–39,525]	18,747 [15,372–22,862]	4.5	2.7	0.56
Brain and central nervous system	18,264 [14,370–23,213]	15,157 [11,539–19,909]	1.9	1.7	0.83
Breast	186,598 [173,041–201,217]	85,787 [77,648–94,779]	40.7	19.4	0.46
Cervix uteri	117,316 [105,999–129,842]	76,745 [68,380–86,133]	25.6	17.7	0.65
Colorectum	66,198	42,875	8.4	5.6	0.65
Corpus uteri	14,024 [10,920–18,010]	4,042 [3,041–5,372]	3.5	1	0.29
Gallbladder	5,454 [3,310–8,988]	4,249 [2,407–7,499]	0.75	0.59	0.78
Hodgkin lymphoma	10,815 [7,994–14,632]	4,315 [3,060–6,086]	0.96	0.43	0.4
Hypo pharynx	2,065 [1,051–4,058]	1,439 [667–3,103]	0.26	0.18	0.7
Kaposi sarcoma	25,010 [20,630–30,320]	13,066 [9,660–17,673]	2.2	1.2	0.52
Kidney	17,718 [13,606–23,073]	10,850 [8,035–14,651]	1.8	1.2	0.61
Larynx	9,908 [7,264–13,515]	6,636 [4,662–9,446]	1.3	0.91	0.67
Leukemia	32,138 [26,882–38,421]	23,891 [19,500–29,271]	3.2	2.6	0.74
Lip, oral cavity	14,286 [11,198–18,225]	8,088 [6,131–10,669]	1.8	1	0.57
Liver	70,542 [61,484–80,935]	66,944 [57,256–78,271]	8.8	8.5	0.95
Lung	45,988 [40,814–51,817.3]	41,171 [35,945–47,157]	6.2	5.6	0.9
Melanoma of skin	6,963 [5,360–9,046]	2,679 [1,989–3,608]	0.9	0.37	0.38
Mesothelioma	1,119 [593–2,110]	1,038 [505–2,135]	0.15	0.14	0.93
Multiple myeloma	8,491 [6,127–11,767]	7,069 [4,877–10,245]	1.1	0.97	0.83
Nasopharynx	10,041 [6,984–14,437]	6,600 [4,367–9,974]	1.1	0.77	0.66
Non–Hodgkin lymphoma	50,516 [43,758–58,317]	30,960 [26,294–36,453]	5.2	3.5	0.61
Esophagus	27,546 [23,474–32,324]	26,097 [21,756–31,305]	3.6	3.4	0.95
Oropharynx	2,913 [1,613–5,262]	1,782 [909–3,491]	0.36	0.23	0.61
Ovary	24,263 [19,547–30,117]	17,008 [13,301–21,748]	5.4	4	0.7
Pancreas	17,070 [13,706–21,260]	16,549 [12,893–21,242]	2.3	2.3	0.97
Penis	2,060 [1,013–4,191]	942 [420–2,113]	0.53	0.25	0.46
Prostate	93,173 [83,906–103,463]	47,249 [41,941–53,228]	29.7	16.3	0.51
Salivary glands	4,920 [2,987–8,104]	2,960 [1,678–5,222]	0.57	0.38	0.6
Stomach	32,402 [26,783–39,200]	27,945 [22,502–34,705]	4.2	3.7	0.86
Testis	3,302 [1,878–5,806]	1,084 [570–2,060]	0.61	0.24	0.33
Thyroid	18,457 [14,616–23,307]	4,443 [3,408–5,793]	2	0.62	0.24
Vagina	2,001 [1,031–3,883]	1,102 [518–2,343]	0.45	0.26	0.55
Vulva	5,144 [3,330–7,946]	2,858 [1,743–4,687]	1.1	0.66	0.56
**All cancers**	**1,109,209 [965,409–1,274,430]**	**711,429 [611,604–827,547]**	**132.1**	**88.8**	**0.64**

*Incidence, all-age new cases; Mortality, all-age deaths; ASIR, age-standardized incidence rate (cases per 100,000); ASMR, age-standardized mortality rate (deaths per 100,000); MIR, mortality-to-incidence ratio. Data source: GLOBOCAN 2020 (International Agency for Research on Cancer). The figures inside square brackets depict 95% uncertainty intervals*.

Males accounted for approximately two-thirds of the liver cancer burden, and male ASIR (11.8/100,000) was nearly twice that of females (6.1/100,000) (Supplementary Table 2). Similarly, colorectal (CRC) cancer was one of the leading cancer groups in Africa, with 66,198 cases with ASIR of 8.4/100,000 and 42,875 deaths with ASMR of 5.6/100,000 (Table 2). CRC was also among the top-10 cancer groups in all 54 countries of Africa (Supplementary Figures 1, 2).

Cancers with the worst MIR were pancreas (0.97), esophagus cancer (0.95), liver (0.95), mesothelioma (0.93), lung (0.90), stomach (0.86), brain and central nervous system (CNS) (0.83), and multiple myeloma (0.83) (Table 2, Figure 3). Among the five most frequent cancer groups, MIR was 0.46 in breast cancer, 0.51 in prostate cancer, 0.65 in CRC and cervical cancer, and 0.95 in liver cancer.

### Age-Specific Distribution of Cancer Burden

**Figure 4:**
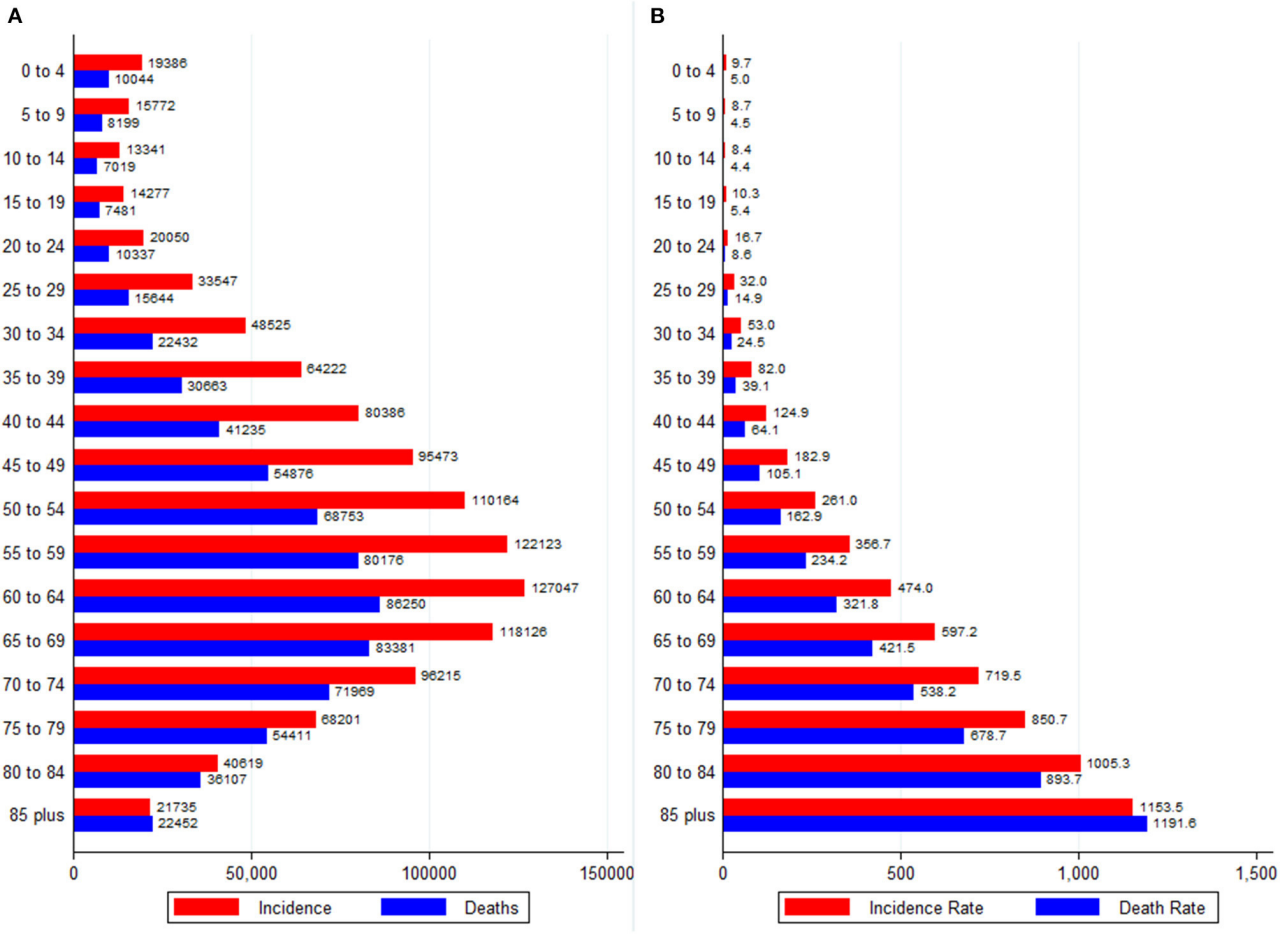
Age-specific burden of all neoplasms in Africa, 2020. **(A)** Absolute numbers and **(B)** Rates per 100,000 people. Data source: GLOBOCAN 2020 (International Agency for Research on Cancer).

Cancer incidence and mortality absolute numbers peaked in the 60–64 age group and declined thereafter (Figure 4A). The incidence and mortality rates (per 100,000), however, increased continuously with age (Figure 4B). The gap between new cancer cases and deaths narrowed with age such that the oldest age groups showed the highest MIR. The variations in the age-specific rates existed across different cancer groups (Supplementary Figure 3). For example, out of all the cancer groups, kidney, leukemia, Hodgkin lymphoma, brain and CNS cancer mainly affected individuals younger than 20 years; in contrast, the burden of CRC, liver, cervical, and ovarian cancer increased up to the age of 69 years, and decreased thereafter (Supplementary Figure 3).

### Association Between Age-Standardized Rates of Cancer and HDI

**Figure 5:**
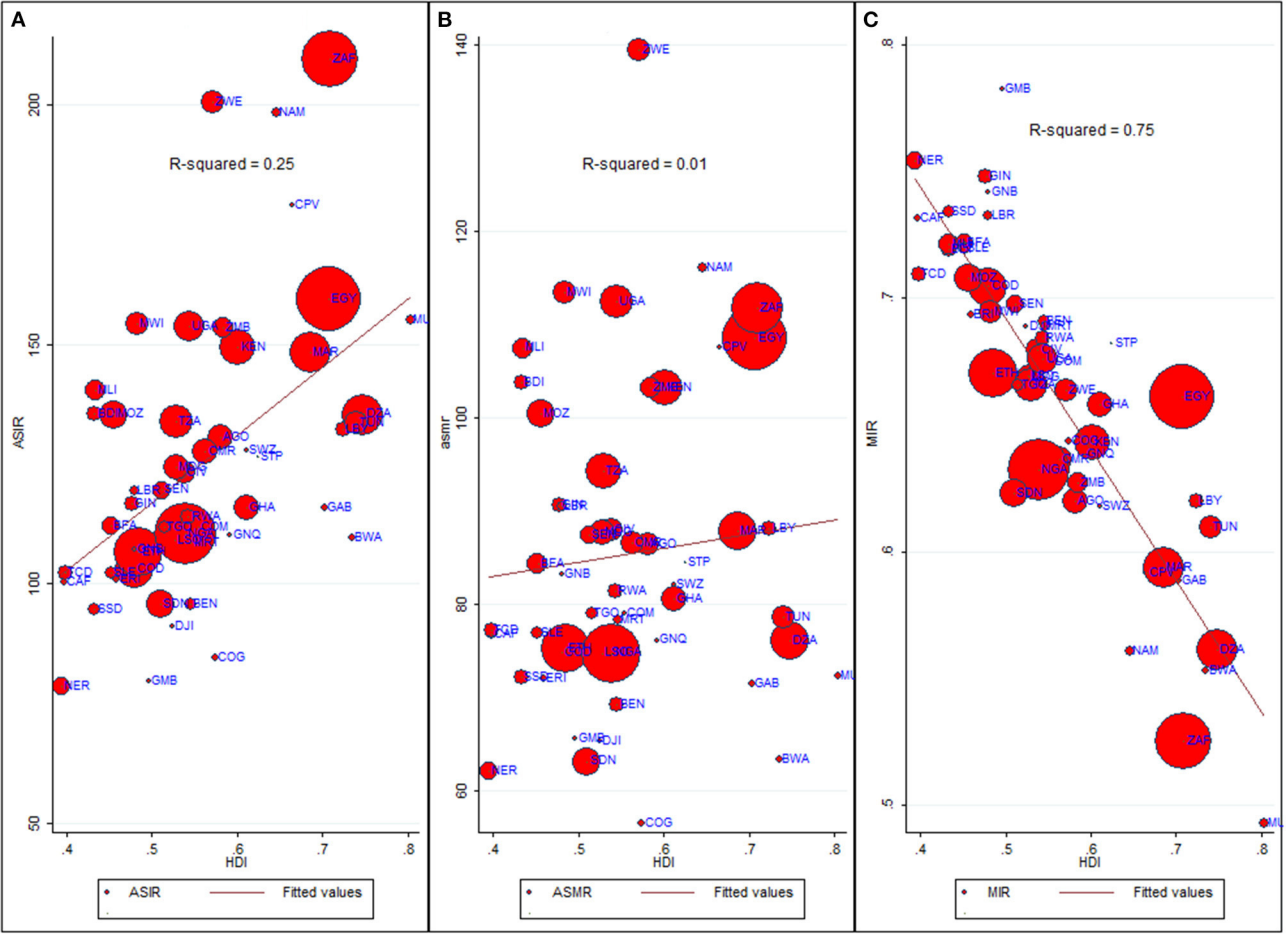
Bivariate relationship between Age-standardized rates, MIR and HDI. ASIR, Age-standardized incidence rate (cases per 100,000); ASMR, Age-standardized mortality rate (deaths per 100,000); MIR, Mortality-to-Incidence Ratio; HDI, Human Development Index. Data source: GLOBOCAN 2020 (International Agency for Research on Cancer). The dots on scatter plot are inflated as per respective ASIR and marked as per country's ISO-3 code. The data of HDI is procured from United Nations Development Program. **(A)** ASIR vs. HDI. **(B)** ASMR vs. HDI. **(C)** MIR vs. HDI.

For all cancer groups combined, the ASIR had a moderately positive relationship with HDI (Pearson's correlation coefficient, *r* = 0.50, *p* < 0.0001, *R*^2^= 0.25); in contrast, the ASMR showed a non-significant relationship with HDI (*r* = 0.09, *p* = 0.52, Figures 5A,B). On the other hand, HDI explained 75% of the variation in MIR (*r* = −0.86, *p* < 0.0001, *R*^2^= 0.75, Figure 5C). Because of a strong relationship between HDI and MIR, we also explored the relationship between MIR of five main cancer groups and HDI. In major cancer groups except liver cancer, HDI explained more than 67% variation in country-level MIR (Supplementary Figure 4). In summary, higher HDI was associated with lower MIR in most of cancer groups.

### Comparison With Other World Regions

The global comparative number of cases and deaths and age-standardized rates for the top 5 neoplasms are shown in Supplementary Figures 5, 6. Although overall Africa had the lowest ASIR (132.1/100,000) than the rest of the continents, its ASMR was similar to the world. For instance, Oceania recorded ASIR of 404.6/100,000 (triple than Africa), yet Oceania (93.2/100,000) had nearly similar ASMR as that of Africa (88.8/100,000) (Supplementary Figure 5). In comparison with other continents, Africa had one of the lowest ASIR (40.7/100,000), but highest ASMR (19.4/100,000) of breast cancer. Similarly, ASIR of CRC was lower in Africa (8.4/100,000) compared to Oceania (29.8/100,000), however the difference in ASMR was quite small between the two regions (Oceania: 9.3/100,000; Africa: 5.6/100,000). Notably, although ASIR of prostate cancer in Africa was one of the lowest (29.7/100,000), the ASMR was the highest (16.3/100,000) among all continents (Supplementary Figure 6).

### Cancer Infrastructure in African Countries

The state of cancer infrastructure in African countries is presented in Supplementary Table 3. Only 16/43 of countries had a cancer policy, strategy, or action plan in place and 28/43 countries had a cancer registry. Even though breast and cervical cancer were the leading malignancies in African females, breast cancer screening *via* mammography was available at the public primary healthcare level in only 15 countries, and even low-cost screening solutions such as clinical breast examination were present in 25 countries. Likewise, cervical cancer screening *via* pap smear test and visual inspection with acetic acid was available in only 32.5% and 25.5% of the African countries, respectively. Screening modalities for CRC were abysmally low with colonoscopy and fecal occult blood test present in only 13.9% and 11.6% of countries, respectively. Africa had the lowest physician density (2.7 per 100,000 population), nurse and midwife density (12.4 per 100,000 population), and radiotherapy units (0.1 per 1 million population) in comparison to global and other WHO regions (Supplementary Table 4).

### Cancer Burden in 2040

**Figure 6:**
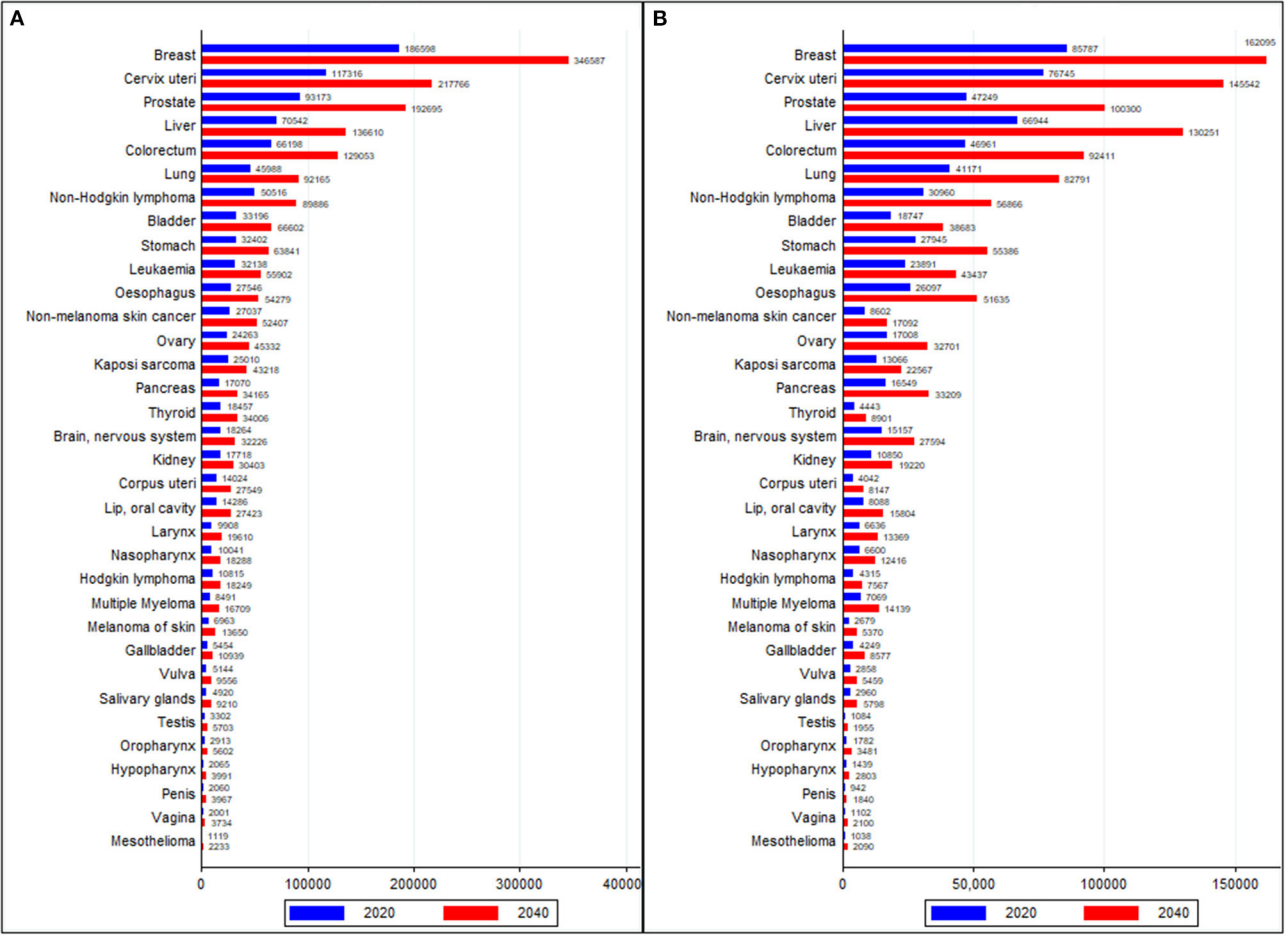
Comparison of cancer burden of all cancer groups in 2020 and forecasted values in 2040 **(A)** Incidence and **(B)** Deaths. Data source: GLOBOCAN 2020 (International Agency for Research on Cancer).

Between 2020 and 2040, the trend of cancer incidence and deaths are forecasted to rise fastest in Africa compared to other world regions. The cancer burden is projected to rise from 1.1 million cases and 711,429 deaths in 2020 to 2.1 million cases and 1.4 million deaths in 2040 in Africa (Supplementary Figure 7). The burden of five major cancer groups (breast, cervical, prostate, CRC, and liver) is expected to double by 2040. By 2040, 12 neoplasms are expected to have more than 50,000 cases per year, whereas cancer deaths are forecasted to be 50,000 or more in nine neoplasms by 2040 in Africa (Figure 6).

## Discussion

We examined the burden of 34 cancer groups in 54 African countries in 2020 using recently released GLOBOCAN 2020 estimates. The cancer incidence count has increased from 715,000 in 2008 (29) to 1.1 million in 2020, and cancer mortality count has increased from 542,000 in 2008 (29) to 711,000 in 2020. In 2020, geographic disparities of ASIR and ASMR were evident in Africa, particularly in main cancer groups. By site, breast, cervical, prostate, liver, and CRC were the major cancer groups accounting for 48% and 45% of new cases and deaths, respectively, in Africa. Egypt, Nigeria, South Africa, Ethiopia, and Morocco had the highest incidence and mortality in 2020; these countries also account for 45% of cases and 44% of cancer deaths. La Reunion had the highest ASIR followed by South Africa, Zimbabwe, Namibia, Cabo Verde and Egypt.

Consistent with previous studies, we found that ASIR was positively associated with HDI (30), whereas MIR exhibited a negative relationship with HDI (25, 31). Although Africa has one of the lowest ASIR, its ASMR is the highest for all neoplasms combined and for individual cancer groups such as breast cancer, prostate cancer, and colorectal cancer. Previous studies have also documented that as the HDI increases, infection-related malignancies (such as cervical cancer, Kaposi sarcoma) are displaced by cancers ascribed to westernization of lifestyle (30). Rapid rise in the incidence of breast, prostate, and CRC have been observed in sub-Saharan African countries (10, 32), with incidence rates of breast and prostate cancer increasing by 3.7% and 5.2% annually respectively, in Kampala, Uganda, from 1991 to 2010 (33). In line with those studies, we found that breast cancer, prostate cancer, and CRC are among the leading malignancies in Africa.

Africa accounted for 19.4% of worldwide cases (117,316 of 604,127) and 22.4% (76,745 of 341,831) of deaths due to cervical cancer along with high ASIR and ASMR (16). We also observed that cervical cancer is the leading malignancy in the majority of sub-Saharan Africa. A previous study also found that women in sub-Saharan Africa were disproportionately affected with higher incidence and concomitant high mortality rates of cervical cancer than other world regions (8). The world health assembly in August 2020 launched a call for Global Strategy for Cervical Cancer elimination as a public health problem by 2030 (34). The strategy was built over three pillars with 90-70-90 target for each country: 90% human papillomavirus (HPV) vaccination coverage of three doses by the age of 15 years, 70% of females of age 15–35 receiving cervical cancer screening, and 90% of females with cervical cancer receiving treatment (34). Modeling studies have shown that HPV vaccination and cervical cancer screening together can help achieve the 90-70-90 target and prevent cervical cancer cases and deaths (35, 36). Bruni et al. (37) reported that only 31% of African countries had included HPV vaccination under national immunization program by 2019, much lower than 85% in America and 77% in Europe. Out of 10 countries for which HPV vaccination coverage (recommended doses) data were available for 2019 in Africa, the vaccination coverage varied from 25% in Senegal to 94% in Rwanda (38).

Breast and cervical cancer are the leading malignancies in African females. Breast cancer was ranked first or second in almost all countries in terms of ASIR. Notably, the ASIRs of breast cancer in Africa are comparable to many high-income countries (16). In African females, 46.2% of cases and 39.2% of deaths occurred in females aged <50 years, consistent with previous findings of young age-profile of breast cancer patients in Africa (9). In addition to younger age profile, late-disease presentation is also quite common in Africa (39), varying from 30% in South Africa to 98% in Nigeria; the stage of disease presentation was also different between black and non-black women in African countries (39). In a Nigerian study (40) with data from 1996 to 2003, there was a mean delay of 11.2 months between onset of symptoms and disease presentation. Out of 297 patients presented with breast cancer between 1996 and 2000 in a tertiary hospital in Uganda, about 75% presented with disease in stage III/IV (41). There are cultural barriers and fear of mastectomy (psychological and actual barriers) in females of several African countries, which leads females hiding tumor from husband or family and often meet a local (traditional) healer before meeting an oncologist. A survey conducted among married women, who had undergone mastectomy in North-western Nigeria revealed that 67.9% felt inadequate as a woman after mastectomy and 38.3% of surveyed women reported being divorced or separated by 3 years of mastectomy (42). Late-disease presentation is further confounded by scarcity of breast conserving surgery due to scarce presence of adjuvant treatments and resources for sentinel lymph node assessment (43).

Prostate cancer is the leading malignancy among male population in Africa with worse mortality rates as well as MIR (i.e., lower 5-year survival rates) than other continents. High incidence and mortality due to prostate carcinoma in African males have been documented before (44, 45). In sub-Saharan Africa, high mortality rates can be attributed to lack of cancer awareness, late presentation and advanced disease, scarcity of pathologists, urologists, radiotherapy, lack of androgen deprivation therapy, limited healthcare access, and low socioeconomic status (43, 44).

Once considered a rare disease in Africa (46), CRC has gained prominence in Africa, with 66,198 cases and 42,875 deaths in 2020. It is now among the top-10 cancer groups in all 54 countries in Africa. With several preventable risk factors, the rising incidence of CRC in Africa can be attributed to the adaptation of western dietary habits and lifestyles (46). Apart from CRC, liver cancer is one of the most lethal malignancies in Africa with more than 70,000 cases and close to 67,000 deaths. The main risk factors of liver cancer pertain to hepatitis B virus (HBV) and hepatitis C viral infection (HCV), aflaxotin exposure, and iron overload (47). In the absence of effective treatment options and resources, countries can work toward reduction in risk factors through HBV vaccination, reduced aflaxotin exposure and alcohol consumption and obesity levels. Although HBV vaccination coverage has increased in majority of African countries, still in 2019, vaccination coverage was 47–99% among children aged <1 year in different African countries (48).

In Africa, there were 62,776 cases and 32,743 deaths due to all cancers in children and adolescents (age <20 years) with leukemia, Hodgkin lymphoma and kidney cancer being the leading cancer groups. There were 4,238 cases and 2,550 deaths due to brain & CNS cancer in children and adolescents. However, the estimates of leukemia, brain & CNS cancer and few other childhood cancers are expected to be severely under-estimated as advanced diagnostic capabilities (e.g., computerized tomography, magnetic resonance imaging and positron emission tomography), immunophenotyping and immunohistopathology to detect and ascertain leukemia and trained staff to categorize these cancers are scarce in Africa (49). In case of leukemia, the flu-like symptoms also prompt patients or their parents to seek help from traditional or local healer which further delays the diagnosis. The absence of population based cancer registries (PBCRs) further confounds the issue. As per Lam et al. (49), the incidence deficit (actual-reported) is much more severe in cancers such as acute leukemia, brain tumors, neuroblastoma, and bone tumors, in which symptoms are less obvious and advanced diagnostic techniques are required. With reference to childhood cancers, therapy abandonment and treatment failures (due to drug toxicity or other comorbid conditions such as infections, malaria and other childhood diseases) are also common in LMICs such as in Africa (50). Distance to oncologic care unit, absence of universal health coverage and high out-of-pocket expenses are the main reasons for therapy abandonment in Africa (49–51).

The low 5-year survival rates (high MIRs) for various malignancies in lower HDI countries owe to late-disease presentation, lack of cancer awareness, and lack of oncologic infrastructure (52, 53). Surgery, radiotherapy and systemic therapies (chemotherapy, targeted therapy, and hormonal therapy) are major treatments paradigms for cancer management and control in Africa (54). However, access to timely, safe, and affordable surgery and radiotherapy is scarce (43, 55). For instance, several African countries have one radiotherapy center per 1 million population against the recommended standard of 1 per 250,000 (56). Additionally, there is a lack of specialized oncology staff, and due to lack of quality training, there is a sparse knowledge among health workers, which results in poor diagnostics and poor referrals to specialized hospitals (53). We also found that Africa lags behind all the WHO regions in terms of physician density, nursing and midwife density and radiotherapy units. The health infrastructure gap between Africa and other resource-rich regions such as Europe is quite large. For instance, physician density of 32.1/100,000 was recorded in Europe, whereas Africa recorded a physician density of only 2.7/100,000. Inadequate health systems have led to a proliferation of traditional medicine including local healers, who does not require radical treatments such as mastectomy and promise cancer treatment and cure without side effects such as hair loss, infertility, erectile dysfunction (43). This further delays the diagnosis and patients often present when palliative care is the only option left, which is also inadequate or unavailable in several sub-Saharan African countries (53). As a category of illness, neoplasms do not receive adequate funding and policymaker's attention due to a significant burden of infectious diseases and a focus on child and maternal health (53). Further, the health policy responses aimed at addressing NCDs have been inadequate and fragmented in the African region (57).

### Study Limitations

Our study had some limitations. First, the GLOBOCAN estimates are generated using data from cancer registries (national or regional) and yet several African countries either do not have PBCRs, or existing registries lack quality or adequate population coverage. Therefore, the estimates reported in this paper are expected to be underreported and downward biased. Nevertheless, uncertainty intervals reported in the study reflect sources of bias such as measurement bias and selection bias due to missing data. Second, the MIR has been considered a proxy indicator of 5-year survival rates. However, it cannot replace actual data collection on cancer survival rates in Africa. We believe that due to lack of actual data on survival rates from PBCRs, MIR can serve as a valuable proxy of comparative 5-year survival rates in different countries. Third, HDI cannot take into account the inequalities that exist within the countries. Fourth, the lag period between exposure to risk factors associated with socioeconomic development and cancer outcomes cannot be taken into consideration because of the cross-sectional nature of our study. Fifth, these estimates are produced for the year 2020, but these estimates do not capture the effect of COVID-19 pandemic on cancer estimates, particularly cancer mortality. More improved estimates reflecting the effects of COVID-19 on cancer incidence and mortality are expected in future iterations of GLOBOCAN. Lastly, the 2040 projections examined in this study are based on the assumption that the risk factors and rates of incidence and mortality remain unchanged by 2040. However, this assumption may not hold true due to changing risk factors including environmental (e.g., air pollution) and behavioral risk factors (e.g., smoking, excessive alcohol consumption, unhealthy diet, physical inactivity and obesity), and increasing life-expectancy. Furthermore, healthcare system's ability to manage the cancer burden is expected to change significantly by 2040. Therefore, the 2040 numbers or rates could be much different than the projections examined in this study.

## Conclusion

Cancer incidence and mortality rates are increasing in Africa and are mirroring global incidence rates in several neoplasms such as breast cancer, prostate cancer, and CRC. Although ASIR of different cancer groups in Africa are substantially lower than other world regions, the mortality rates are comparable or higher than other regions. We estimated that between 2020 and 2040, the trend of cancer incidence and deaths are forecasted to rise the fastest in Africa compared to other world regions. Nearly 2.1 million cases and 1.4 million deaths are projected by 2040 in Africa; this, however, can be even higher due to escalation of behavioral and environmental risk factors, increasing life-spans, and improvements in cancer registration. However, due to lack of resources as evidenced by low HDI, only a few African countries have a strong policy focused on cancer control and management. Cancer control and mitigation strategies in Africa demand a holistic approach that includes vaccination (HPV, Hepatitis B virus, Epstein-Barr Virus), prevention of risk factors (e.g., smoking, alcohol, air pollution, and diet), raising awareness, building capacity (e.g., cancer registry coverage and quality, cancer specialists, and support staff), and adoption of cost-effective diagnostic, therapeutic, and palliative care.

## Data Availability Statement

The original contributions presented in the study are included in the article/Supplementary Material, further inquiries can be directed to the corresponding author.

## Author Contributions

RS wrote the manuscript, did data analysis, and the guarantor for this work. CF and PSs performed data analysis and interpreted results. PSs wrote the manuscript and supervised the work. A, MN, PSe, AES, NDA, JO, FA-M, and PSs reviewed the manuscript and approved the final version. All authors contributed to the article and approved the submitted version.

## Conflict of Interest

The authors declare that the research was conducted in the absence of any commercial or financial relationships that could be construed as a potential conflict of interest.

## Publisher's Note

All claims expressed in this article are solely those of the authors and do not necessarily represent those of their affiliated organizations, or those of the publisher, the editors and the reviewers. Any product that may be evaluated in this article, or claim that may be made by its manufacturer, is not guaranteed or endorsed by the publisher.
