# Supplementary material for: Mapping Cancer in Africa: A Comprehensive and Comparable Characterization of 34 Cancer Types Using Estimates From GLOBOCAN 2020
Source: Front Public Health. 2022 Apr 25;10:839835. doi: 10.3389/fpubh.2022.839835 (PMC9082420; doi:10.3389/fpubh.2022.839835)
Supplement: Supplementary file 1 [file Data_Sheet_1.pdf]

Supplementary Table 1 Cancer Groups and ICD-10 Classification

**Cancer**	**ICD**
Bladder	C67
Brain and Central Nervous System	C70-72
Breast	C50
Cervix Uteri	C53
Colorectum	C18-21
Corpus Uteri	C54
Gallbladder	C23-24
Hodgkin Lymphoma	C81
Hypopharynx	C12-13
Kaposi sarcoma	C46
Kidney	C64-65
Larynx	C32
Leukemia	C91-95
Lip and Oral Cavity	C00-06
Liver	C22
Lung	C33-34
Skin Melanoma	C43
Mesothelioma	C45
Multiple Myeloma	C88+C90
Nasopharynx	C11
Non-Hodgkin Lymphoma	C82-86, C96
Oesophagus	C15
Oropharynx	C09-10
Ovary	C56
Pancreas	C25
Penis	C60
Prostate	C61
Salivary Glands	C07-08
Stomach	C16

**Cancer**	**ICD**
Testis	C62
Thyroid	C73
Vagina	C52
Vulva	C51
**All Cancers**	C00-97

Supplementary Table 2 Sex-specific Burden of 34 Cancer Groups in Africa, 2020

	Males					Females				
**Cancer**	**Incidence**	**Mortality**	**ASIR**	**ASMR**	**MIR**	**Incidence**	**Mortality**	**ASIR**	**ASMR**	**MIR**
**All cancers**	**475,753 [386,823-585,128]**	**323,883 [258,540-405,740]**	**126.8**	**90.4**	**0.68**	**633,456 [525,277-763,915]**	**387,546 [316,060-475,200]**	**139.5**	**89.2**	**0.61**
Bladder	23,996 [19,641-29,316]	13,502 [10,752-16,956]	7.2	4.4	0.56	9,200 [6,447-13,129]	5,245 [3,500-7,860]	2.3	1.3	0.57
Brain and central nervous system	9,666 [6,989-13,367]	8,058 [5,573-11,651]	2.1	1.9	0.83	8,598 [6,021-12,278]	7,099 [4,734-10,646]	1.7	1.5	0.83
Breast						186,598 [173,041-201,217]	85,787 [77,648-94,779]	40.7	19.4	0.46
Cervix Uteri						117,316 [105,999-129,842]	76,745 [68,380-86,133]	25.6	17.7	0.65
Colorectum	34,060	22,046	9.4	6.3	0.65	32,138	20,829	7.6	5	0.65
Corpus Uteri						14,024 [10,920-18,010]	4,042 [3,041-5,372]	3.5	1	0.29
Gallbladder	1,823 [762-4,358]	1,410 [523-3,800]	0.55	0.43	0.77	3,631 [1,974-6,680]	2,839 [1,419-5,678]	0.91	0.72	0.78
Hodgkin lymphoma	6,386 [4,300-9,483]	2,610 [1,665-4,092]	1.2	0.53	0.41	4,429 [2,771-7,079]	1,705 [1,000-2,906]	0.78	0.33	0.38
Hypo pharynx	1,353 [578-3,168]	965 [367-2,539]	0.36	0.26	0.71	712 [234-2,164]	474 [134-1,678]	0.17	0.11	0.67
Kaposi sarcoma	16,192 [12,624-20,769]	8,472 [5,971-12,021]	3	1.6	0.52	8,818 [6,509-11,947]	4,594 [2,525-8,358]	1.5	0.78	0.52
Kidney	9,621 [6,782-13,649]	5,848 [3,929-8704]	2.1	1.4	0.61	8,097 [5,412-12,114]	5,002 [3,163-7,909]	1.5	0.98	0.62
Larynx	8,486 [6,079-11,845]	5,675 [3,883-8,293.2]	2.4	1.7	0.67	1,422 [608-3,325]	961 [366-2,525]	0.33	0.23	0.68
Leukaemia	17,841 [14,044-22,665]	13,262 [10,102-17,411]	3.7	3.1	0.74	14,297 [10,933-18,696]	10,629 [7,834-14,421]	2.8	2.2	0.74
Lip, oral cavity	8,174 [5,994-11146]	4,606 [3,237-6,554]	2.1	1.3	0.56	6,112 [4,125-9,056]	3,482 [2,227-5,445]	1.4	0.85	0.57
Liver	45,142 [38,258-53,265]	42,740 [35,408-51,590]	11.8	11.3	0.95	25,400 [19,845-32,510]	24,204 [18,280-32,048]	6.1	5.9	0.95
Lung	33,282 [28,832-38,418]	29,760 [25,277-35,037]	9.8	8.9	0.89	12,706 [10,249-15,751]	11,411 [89,37-14,570]	3.2	2.9	0.9

Melanoma of skin	3,111 [2,153-4,496]	1,193 [785-1,813]	0.88	0.36	0.38	3,852 [2,655-5,588]	1,486 [973-2,269]	0.93	0.37	0.39
Mesothelioma	765 [360-1,624]	702 [298-1,653]	0.22	0.21	0.92	354 [109-1,148]	336 [88-1,281]	0.09	0.09	0.95
Multiple myeloma	4,537 [2,892-7,117]	3,756 [2,251-6,268]	1.3	1.1	0.83	3,954 [2,463-6,348]	3,313 [1,934-5,676]	0.98	0.85	0.84
Nasopharynx	6,527 [4,162-10,236]	4,278 [2,564-7,137]	1.5	1.1	0.66	3,514 [1,900-6,498]	2,322 [1,154-4,672]	0.7	0.51	0.66
Non-Hodgkin lymphoma	27,892 [23,021-33,794]	16,943 [13,620-21,077]	6	4	0.61	22,624 [18,221-28,091]	14,017 [10,958-17,929]	4.6	3	0.62
Oesophagus	15,034 [12,231-18,480]	14,286 [11,297-18,065]	4.2	4	0.95	12,512 [9,713-16,117]	11,811 [8,856-15,753]	3.1	2.9	0.94
Oropharynx	2,164 [1,094-4,281]	1,346 [620-2,924]	0.57	0.37	0.62	749 [229-2,452]	436 [113-1,679]	0.17	0.1	0.58
Ovary						24,263 [19,547-30,117]	17,008 [13,301-21,748]	5.4	4	0.7
Pancreas	9,239 [6,860-12,443]	8,936 [6,369-12,537]	2.7	2.6	0.97	7,831 [5,659-10,837]	7,613 [5,261-11,016]	2	1.9	0.97
Penis	2,060 [1,013-4,191]	942 [420-2,113]	0.53	0.25	0.46			0	0	
Prostate	93,173 [83,906-103,463]	47,249 [41,941-53,228]	29.7	16.3	0.51			0	0	
Salivary glands	2,654 [1,340-5,258]	1,605 [737-3,493]	0.68	0.47	0.6	2,266 [1,092-4,703]	1,355 [590-3,109]	0.48	0.32	0.6
Stomach	17,500 [13,621-22,484]	15,099 [11,354-20,079]	4.9	4.3	0.86	14,902 [11,116-19,977]	12,846 [9,204-17,928]	3.6	3.2	0.86
Testis	3,302 [1,878-5,806]	1,084 [570-2,060]	0.61	0.24	0.33			0	0	
Thyroid	3,839 [2,342-6,293]	1,106 [630-1,940]	0.94	0.34	0.29	14,618 [11,219-19,047]	3,337 [2,456-4,533]	3.1	0.85	0.23
Vagina						2,001 [1,031-3,883]	1,102 [518-2,343]	0.45	0.26	0.55
Vulva						5,144 [3,330-7,946]	2,858 [1,743-4,687]	1.1	0.66	0.56

Incidence: All-age new cases; Mortality: All-age deaths; ASIR: Age-standardized incidence rate (cases per 100 000); ASMR: Age-standardized mortality rate (deaths per 100 000); MIR: Mortality-to-incidence ratio. Data Source: GLOBOCAN 2020 (International Agency for Research on Cancer). The figures inside square brackets depict 95% uncertainty intervals.

Supplementary Table 3 Cancer Infrastructure of African Countries

			Cervix Uteri		Breast Cancer		Colorectal Cancer	
**Country**	**Cancer policy/ strategy/ action plan**	**Cancer Registry**	**PAP**	**VIA**	**CBE**	**Mammogram**	**FOBT**	**Colonoscopy**
Algeria	1	1	1	1	1	1	0	0
Angola								
Benin	0	0	0	0	1	1	0	0
Botswana	0	1	1	0	0	1	0	0
Burkina Faso	0	1	1	0	1	1	0	1
Burundi	0	0	0	0	0	0	0	0
Cameroon	1	1	1	1	1	0	1	0
Cape Verde								
Central African Republic	0	0	1	0	1	0	0	0
Chad								
Comoros	0	0	1	0	1	0	1	0
Republic of the Congo	1	1	1	1	1	1	0	1
Cote d'Ivoire	1	1	0	0	0	0	0	0
The Democratic Republic of the Congo								
Djibouti	0	0	0	0	1	1	0	0
Egypt	0	1	0	1	1	0	1	0
Equatorial Guinea	0	0	0	DK	1	0	0	0

			Cervix Uteri		Breast Cancer		Colorectal Cancer	
**Country**	**Cancer policy/ strategy/ action plan**	**Cancer Registry**	**PAP**	**VIA**	**CBE**	**Mammogram**	**FOBT**	**Colonoscopy**
Eritrea	1	0	0	DK	0	0	0	0
Eswatini	0	1	1	1	1	0	0	0
Ethiopia								
Gabon	0	1	0	0	1	1	1	1
Ghana	1	1	0	0	0	0	0	0
Guinea	1	1	0	0	0	0	0	0
Guinea-Bissau	0	0	0	0	1	0	0	0
Kenya	1	1	0	0	1	0	0	0
Lesotho	0	0	0	0	0	0	0	0
Liberia	0	1	0	0	1	1	0	0
Libya	0	0	0	0	0	0	0	0
Madagascar	1	1	0	0	0	0	0	0
Malawi	0	1	0	1	0	0	0	0
Mali	DK	1	1	1	1	1	0	0
Mauritania	1	0	0	0	0	0	0	0
Mauritius								
Morocco	1	1	1	1	1	1	0	1
Mozambique	1	1	0	1	1	0	0	0
Namibia	DK	1	1	0	1	1	1	DK
Niger	0	1	0	0	1	0	0	0

			Cervix Uteri		Breast Cancer		Colorectal Cancer	
**Country**	**Cancer policy/ strategy/ action plan**	**Cancer Registry**	**PAP**	**VIA**	**CBE**	**Mammogram**	**FOBT**	**Colonoscopy**
Nigeria	0	1	0	0	0	0	0	0
Rwanda	1	0	0	0	0	0	0	0
Sao Tome and Principe	0	0	1	0	0	0	0	0
Senegal	0	0	1	1	1	1	0	1
Sierra Leone								
Somalia	0	0	0	0	1	0	0	0
South Africa								
South Sudan								
Sudan	1	1	0	0	0	0	0	0
Tanzania								
The Gambia	0	1	1	1	0	1	0	0
Togo	1	1	0	0	0	0	0	0
Tunisia	0	1	0	0	1	1	0	0
Uganda	0	1	0	0	1	1	0	0
Zambia	1	1	0	0	0	0	0	0
Zimbabwe	0	1	0	0	1	0	0	1
**TOTAL**	**16/43**	**28/43**	**14/43**	**11/43**	**25/43**	**15/43**	**5/43**	**6/43**

Data Source: All the data is procured from WHO Cancer Country Profiles. The answer to the question: Cancer policy/ strategy/ action plan and cancer registry were responded as yes (1) or no (0). The answer to the question on screening availability was coded as: generally available at the public primary

health care level (1) and not generally available at the public primary health care level (0). DK stands for don't know. The countries without entry did not respond to the survey. PAP: pap smear; VIA: Visual inspection with acetic acid; CBE: Clinical breast exam; FOBT: Fecal occult blood test.

Supplementary Table 4 Comparison of Cancer Infrastructure in Africa with other World Health Organization Regions

**Region**	**Physician Density (per 100,000 population)**	**Nursing and Midwife Density (per 100,000 population)**	**Radiotherapy Units (per 1 million population)**
**Global**	**13.9**	**28.6**	**1.8**
Europe	32.1	80.2	3.9
Americas	21.5	44.9	5.3
Western Pacific	15.5	26.9	--
East Mediterranean	12.7	18.0	0.4
South-East Asia	5.9	15.3	0.3
Africa	2.7	12.4	0.1

Data Source: Atlas of African Health Statistics 2016 (WHO).

Supplementary Figures

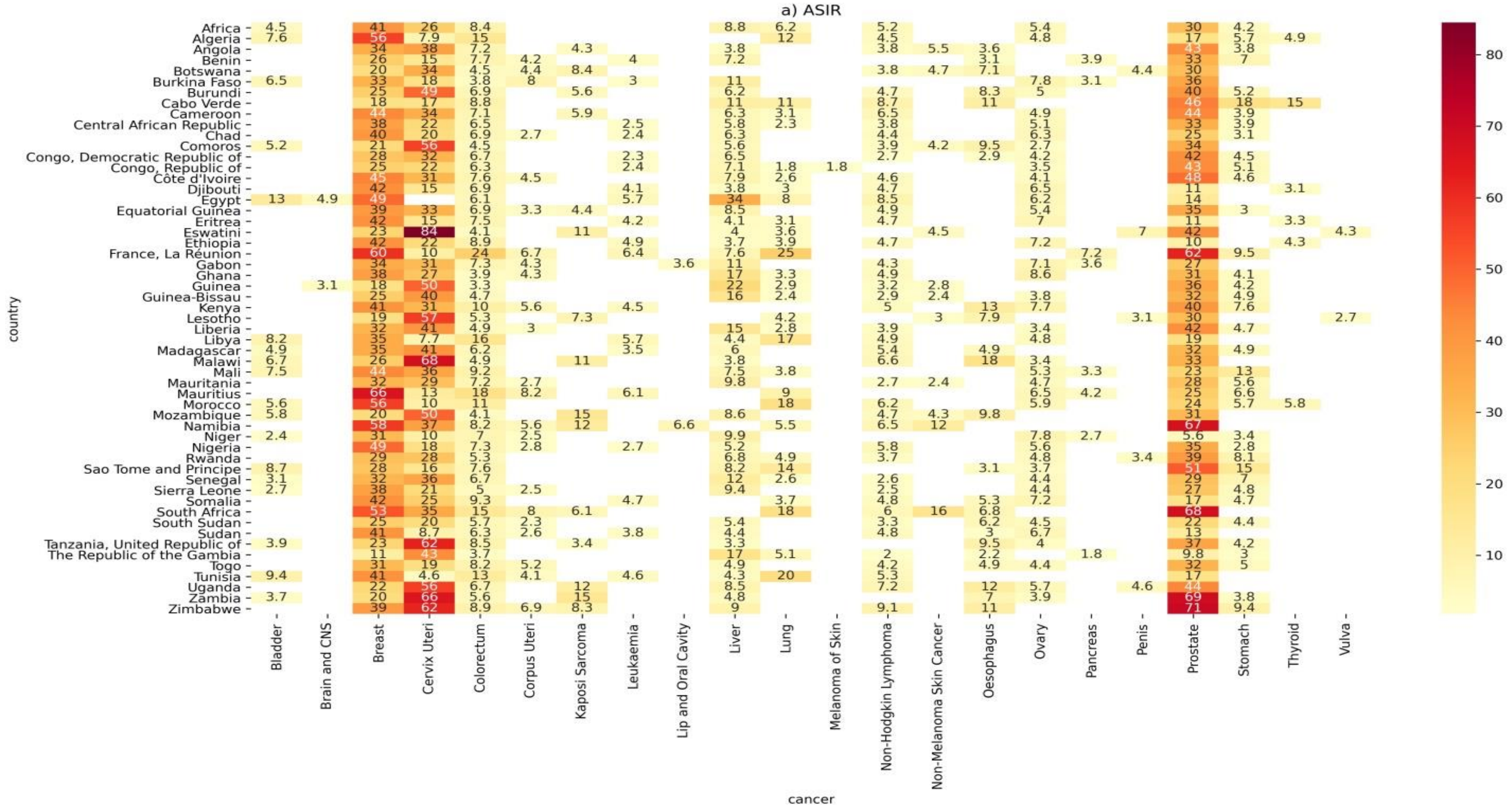

Supplementary Figure 1 Top-ten Cancer Groups in 54 African Countries in terms of ASIR.

The cancer groups in a country were ranked as per age-standardised incidence rate (ASIR) in the given country. The numbers in the cells represent the ASIR of a particular cancer group in that country. The color of the cell represents the ASIR, also varying from yellow (low) to dark red (very high), as indicated in the legend bar on the right.

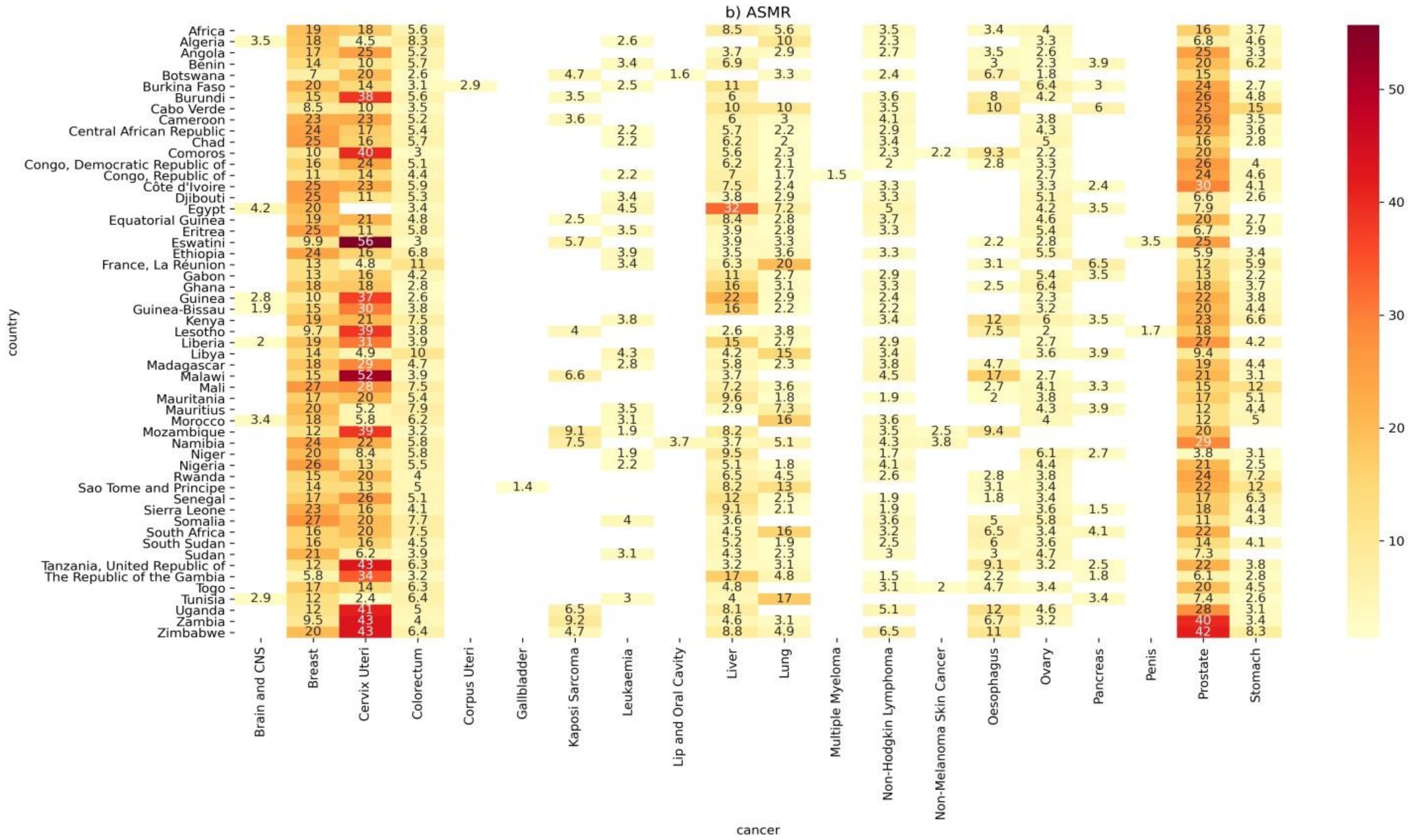

Supplementary Figure 2 Top-ten Cancer Groups in 54 African Countries in terms of ASMR.

The cancer groups in a country were ranked as per the age-standardized mortality rate (ASMR) in the given country. The numbers in the cells represent the ASMR of a particular cancer group in that country. The cell color represents the ASMR, also varying from yellow (low) to dark red (very high), as indicated in the legend bar on the right. Data Source: GLOBOCAN 2020 (International Agency for Research on Cancer).

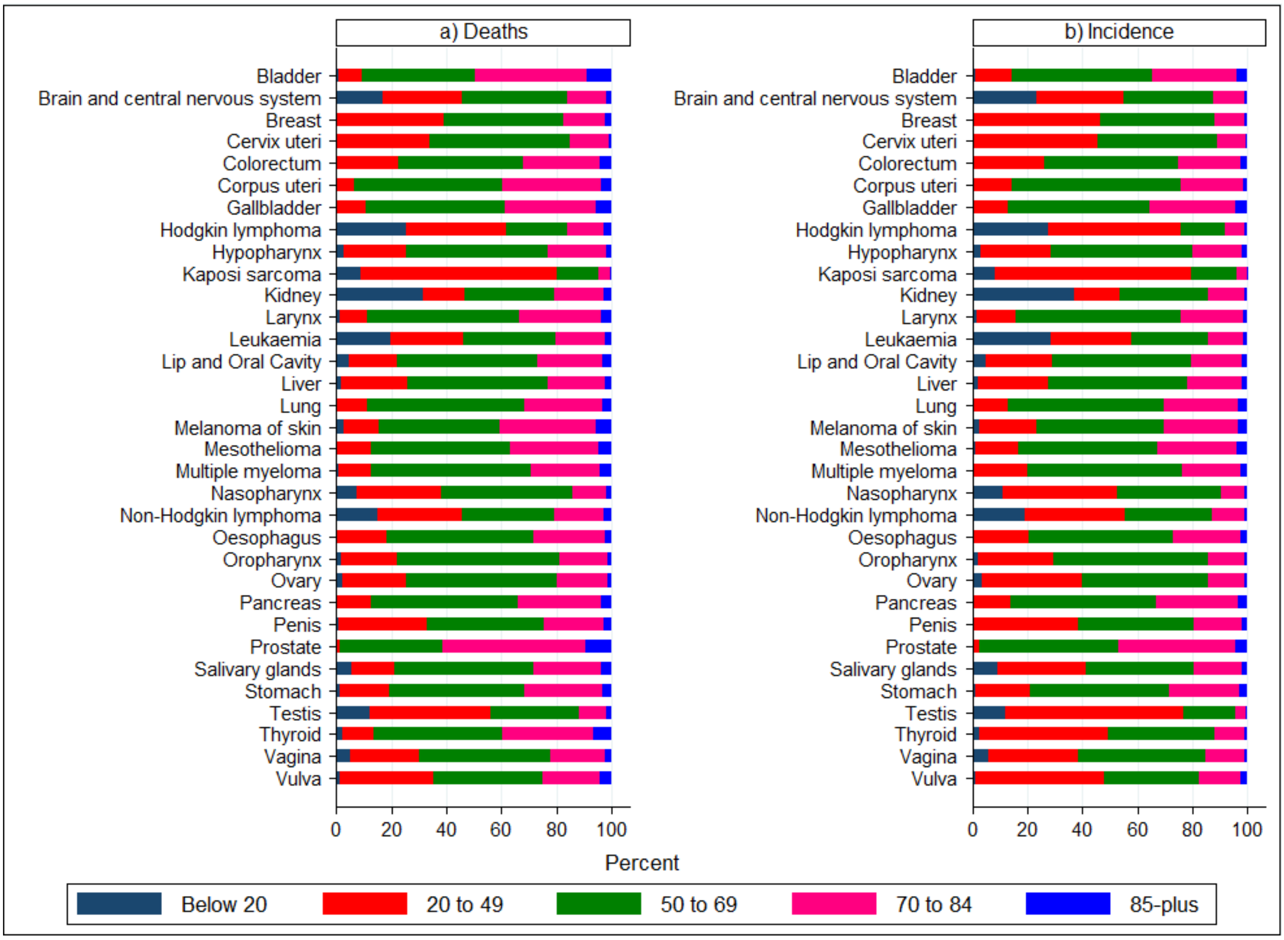

Supplementary Figure 3 Age-Group wise Burden of 34 Cancer Groups in Africa, 2020 a) Deaths b) Incidence.

Incidence: Age-group wise cases; Deaths: Age-group wise deaths. Data Source: GLOBOCAN 2020 (International Agency for Research on Cancer).

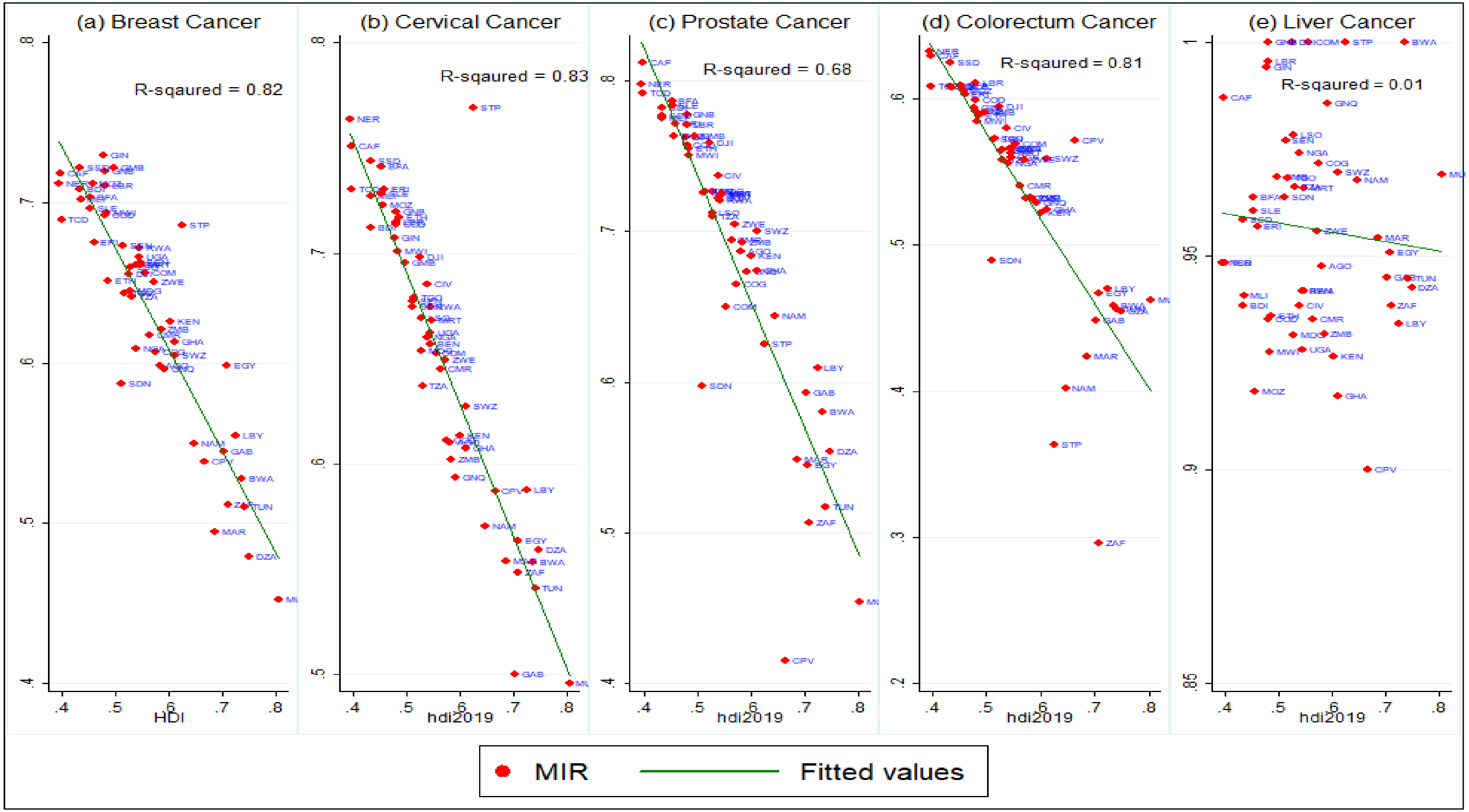

Supplementary Figure 4 Association between HDI and MIR for the Top-5 Cancer Groups. a) Breast Cancer b) Cervical Cancer c) Prostate Cancer d) Colorectum Cancer e) Liver Cancer. The dots on the scatter plot are marked as per the country's ISO-3 code. MIR: Mortality-to-Incidence Ratio; HDI: Human Development Index. Data Source: GLOBOCAN 2020 (International Agency for Research on Cancer). The data about HDI is procured from United Nations Development Program.

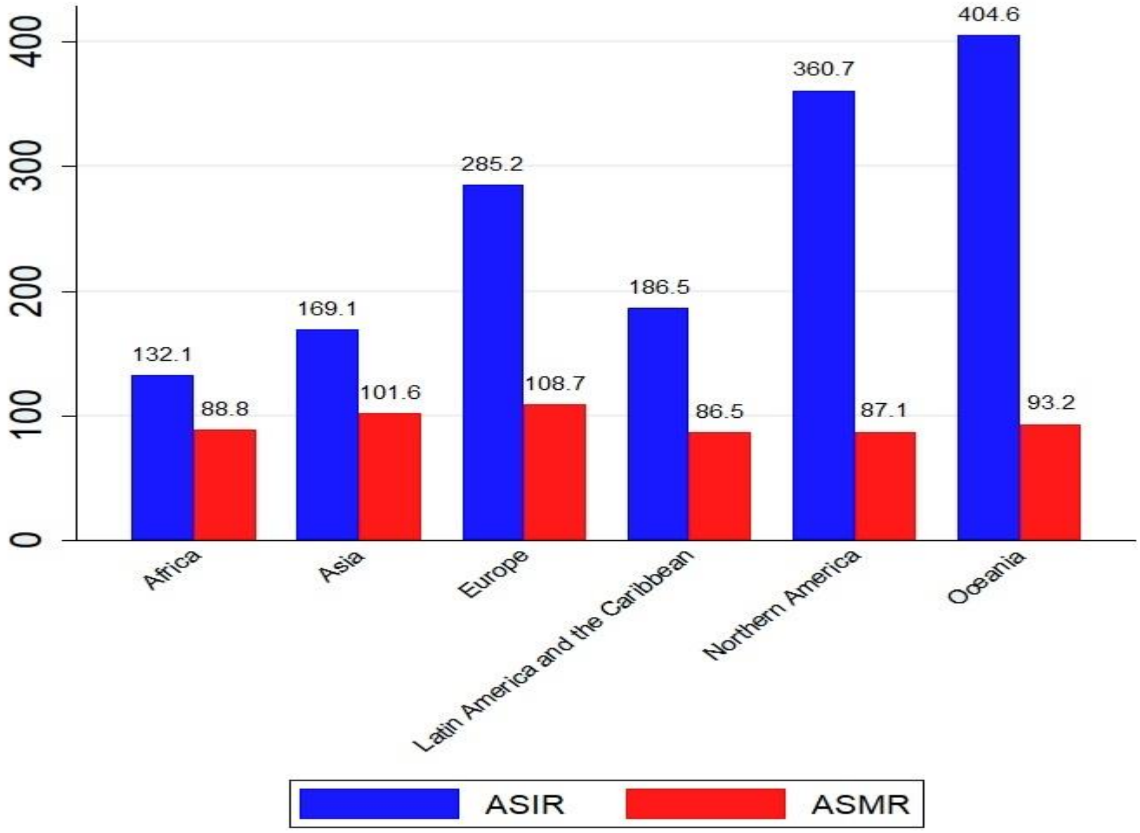

Supplementary Figure 5 Comparison of Africa with Other Continents in terms of Age-standardized Rates.

ASIR: Age-standardized incidence rate (cases per 100 000); ASMR: Age-standardized mortality rate (deaths per 100 000). Data Source: GLOBOCAN 2020 (International Agency for Research on Cancer)

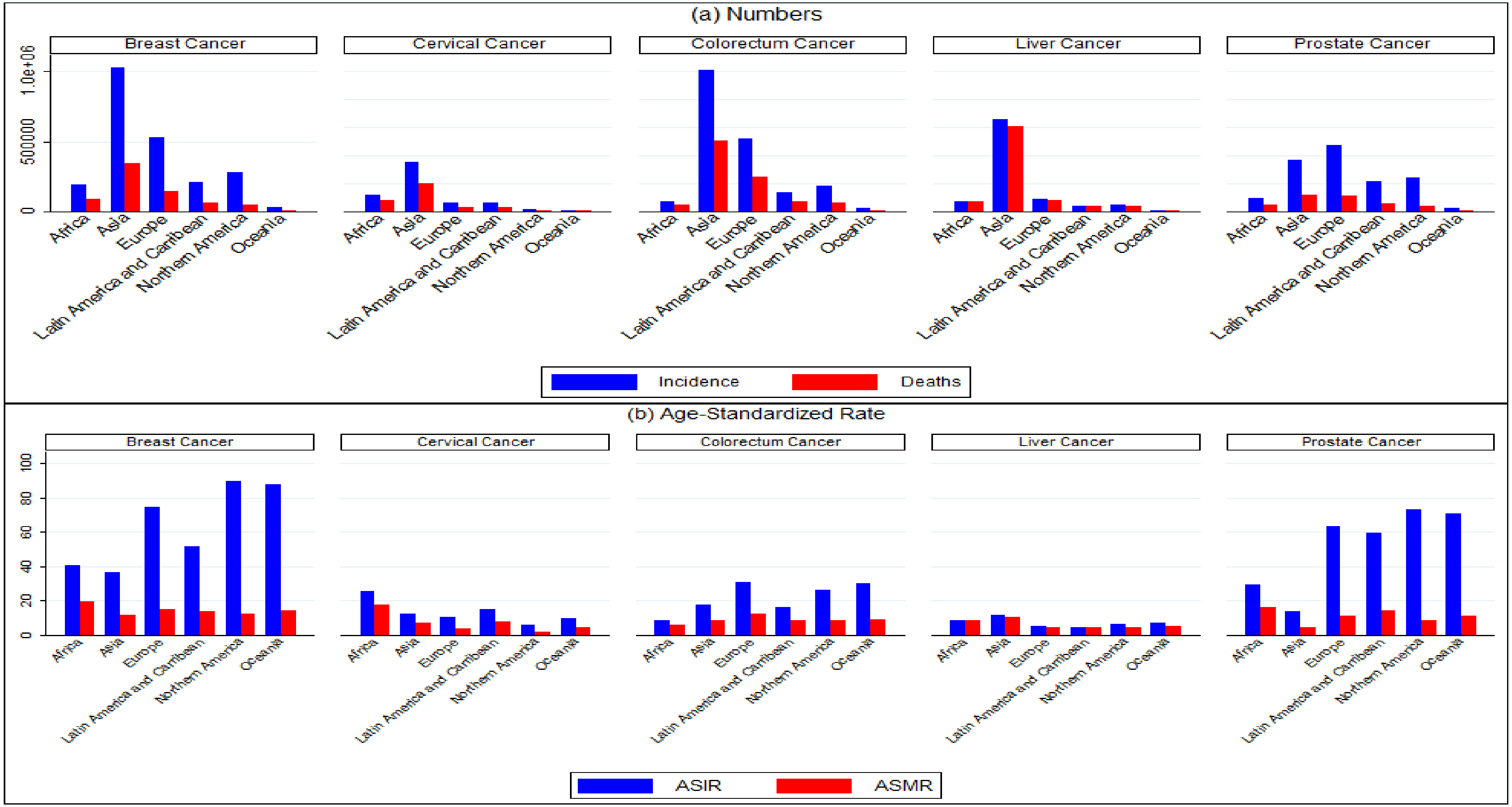

Supplementary Figure 6 Comparison of Africa with Other Continents in terms of Top-5 Cancer Groups. a) Numbers b) Age-Standardized Rates.

Incidence: All-age new cases; Deaths: All-age deaths; ASIR: Age-standardized incidence rate (cases per 100 000); ASMR: Age-standardized mortality rate (deaths per 100 000); MIR: Mortality-to-incidence ratio. Data Source: GLOBOCAN 2020 (International Agency for Research on Cancer).

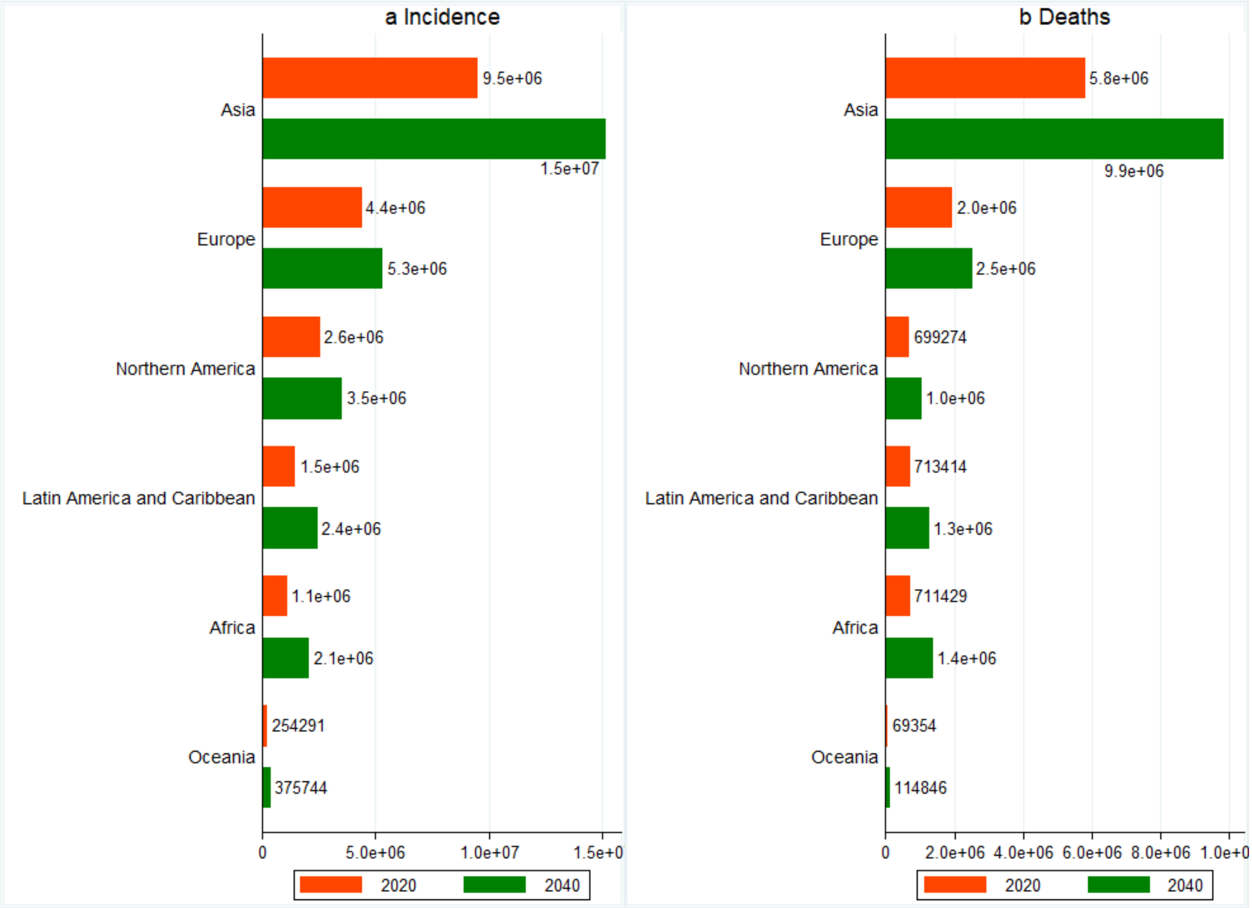

Supplementary Figure 7 Comparison of Cancer Burden in Africa with other WHO Regions in 2020 and Forecasted Values in 2040. a) Incidence b) Deaths**.**

Data Source: GLOBOCAN 2020 (International Agency for Research on Cancer).
